# Optimal Heart Rate Control Improves Long-Term Prognosis of Decompensated Heart Failure with Reduced Ejection Fraction

**DOI:** 10.3390/medicina59020348

**Published:** 2023-02-12

**Authors:** Ming-Lung Tsai, Shu-I Lin, Yu-Cheng Kao, Hsuan-Ching Lin, Ming-Shyan Lin, Jian-Rong Peng, Chao-Yung Wang, Victor Chien-Chia Wu, Chi-Wen Cheng, Ying-Hsiang Lee, Ming-Jui Hung, Tien-Hsing Chen

**Affiliations:** 1 Division of Cardiology, Department of Internal Medicine, New Taipei Municipal TuCheng Hospital, New Taipei 236, Taiwan; 2 College of Medicine, Chang Gung University, Taoyuan 333, Taiwan; 3 Cardiovascular Center, MacKay Memorial Hospital, Taipei 104, Taiwan; 4 Department of Medicine, MacKay Medical College, New Taipei 252, Taiwan; 5 Department of Nursing, MacKay Junior College of Medicine, Nursing, and Management, Taipei 112, Taiwan; 6 Division of Cardiology, Heart Center, Cheng Hsin General Hospital, Taipei 112, Taiwan; 7 Division of Cardiology, Department of Internal Medicine, Chang Gung Memorial Hospital at Keelung, Keelung 204, Taiwan; 8 Division of Cardiology, Department of Internal Medicine, Chang Gung Memorial Hospital at Chiayi, Chiayi 613, Taiwan; 9 Division of Cardiology, Department of Internal Medicine, Chang Gung Memorial Hospital at Linkou, Taoyuan 333, Taiwan; 10 Department of Artificial Intelligence and Medical Application, MacKay Junior College of Medicine, Nursing, and Management, Taipei 112, Taiwan

**Keywords:** heart rate, heart failure, mortality

## Abstract

*Background and Objectives:* An elevated heart rate is an independent risk factor for cardiovascular disease; however, the relationship between heart rate control and the long-term outcomes of patients with heart failure with reduced ejection fraction (HFrEF) remains unclear. This study explored the long-term prognostic importance of heart rate control in patients hospitalized with HFrEF. *Materials and Methods*: We retrieved the records of patients admitted for decompensated heart failure with a left ventricular ejection fraction (LVEF) of ≤40%, from 1 January 2005 to 31 December 2019. The primary outcome was a composite of cardiovascular death or hospitalization for heart failure (HHF) during follow-up. We analyzed the outcomes using Cox proportional hazard ratios calculated using the patients’ heart rates, as measured at baseline and approximately 3 months later. The mean follow-up duration was 49.0 ± 38.1 months. *Results*: We identified 5236 eligible patients, and divided them into five groups on the basis of changes in their heart rates. The mean LVEFs of the groups ranged from 29.1% to 30.6%. After adjustment for all covariates, the results demonstrated that lesser heart rate reductions at the 3-month screening period were associated with long-term cardiovascular death, HHF, and all-cause mortality (*p* for linear trend = 0.033, 0.042, and 0.003, respectively). The restricted cubic spline model revealed a linear relationship between reduction in heart rate and risk of outcomes (*p* for nonlinearity > 0.2). *Conclusions*: Greater reductions in heart rate were associated with a lower risk of long-term cardiovascular death, HHF, and all-cause mortality among patients discharged after hospitalization for decompensated HFrEF.

## 1. Introduction

A high resting heart rate is an independent risk factor for all-cause mortality, cardiovascular mortality, and cardiovascular events among the general population [1,2] as well as among patients with cardiovascular disease, coronary artery disease, hypertension, heart failure, and diabetes [3,4,5,6,7,8,9]. The relationship between heart rate and adverse outcomes may be mediated by the effects of heart rate on coronary blood flow, cardiac contractility, and energy expenditure [7,10]. Reducing a patient’s heart rate can reduce afterload, relieve left ventricular wall stress, and increase the stroke volume of the left ventricle, thus improving the patient’s heart function and alleviating their cardiovascular symptoms [11]. These findings suggest that physicians should implement interventions to reduce the heart rates of patients with HFrEF and improve their clinical outcomes. 

Numerous studies have explored the effects of heart rate control on patients with heart failure. A randomized controlled trial involving patients with HFrEF, the Ivabradine and Outcomes in Chronic Heart Failure (SHIFT) study, demonstrated that reductions in heart rate due to ivabradine benefit patients with HFrEF who have heart rates of >70 bpm, despite receiving guideline-directed therapies, including beta blockers [12]. The rates of major adverse cardiovascular events, namely hospitalization for heart failure (HHF) and cardiovascular death, were significantly lower in the ivabradine group than in the placebo group, especially among the patients with higher baseline heart rates. 

The importance of heart rate monitor and control have been addressed in major guidelines [13,14]; however, the relationship between heart rate reductions and health outcomes have not been thoroughly evaluated. In addition, few studies have analyzed the long-term outcomes of heart rate control for patients discharged after hospitalization for decompensated HFrEF. We conducted this study to evaluate the effect of heart rate reductions on the long-term outcomes of patients with HFrEF discharged from the hospital through an analysis of records from multiple healthcare institutions.

## 2. Method

### 2.1. Data Source

This study was conducted using the Chang Gung Research Database (CGRD), a de-identified database managed by the largest healthcare provider in Taiwan, the Chang Gung Memorial Hospital (CGMH) healthcare system. The CGMH system is multi-institutional, comprising seven healthcare institutions (four tertiary academic medical centers and three teaching hospitals) across Taiwan. The use of data from the CGRD as the basis for accurate estimates in medical studies has been validated [15]. The Chang Gung Memorial Hospital Institutional Review Board approved this study and waived the requirement for informed consent. The patients’ records were anonymized and de-identified before analysis. For data generated before 2015, we used the *International Classification of Diseases*, *Ninth Revision, Clinical Modification* (*ICD*-*9-CM*) for diagnosis, whereas for data generated after 2016, we used both the *ICD*-*9-CM* and the *ICD Tenth Revision* (*ICD-10-CM*). More information regarding the CGRD has been published in other articles [15,16]. This study was conducted in accordance with the principles outlined in the Declaration of Helsinki [17].

### 2.2. Study Group and Cohort

**Figure 1:**
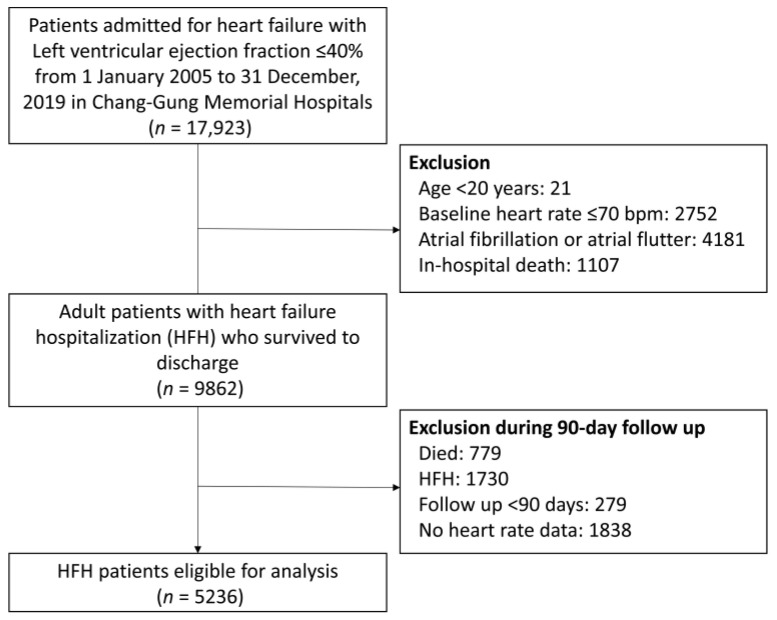
Flowchart of patient inclusion and exclusion.

From the CGRD, we retrieved the records of patients admitted for decompensated heart failure with a left ventricular ejection fraction (LVEF) of ≤40%, from 1 January 2005 to 31 December 2019. The index date was the date when each patient was discharged after index heart failure admission. Each patient’s LVEF was determined on the basis of the echocardiography report generated during the index admission. Each patient’s baseline heart rate was defined as their first heart rate recorded after the index admission. The first recorded heart rate at admission is the condition before medications or treatments for heart failure control. Each patient’s follow-up heart rate was defined as their heart rate recorded at the 3-month screening period in the outpatient department. Clinically, physicians may frequently adjust the medication and treatment for a short period after discharge. It was noted that the medications prescribed for heart failure were less changed until a period of 2–4 months after discharge. Thus, we chose the 3 months after discharge as the screening period. Patients were excluded if they were aged younger than 20 years, had a baseline heart rate of <70 bpm, had a diagnosis of atrial fibrillation or atrial flutter before or during the index admission, or did not survive to discharge. Patients who died, presented with heart failure exacerbation and required readmission before the 3-month screening period, had follow-up periods of <90 days, or lacked follow-up heart rate measurements, were also excluded (Figure 1). A total of 5236 patients with decompensated heart failure and an LVEF of ≤40% requiring hospitalization with follow-up durations of over 3 months were determined to be eligible for inclusion.

### 2.3. Covariate Measurements

The covariates of interest were demographic characteristics (age, sex, smoking status, and body mass index), baseline vital signs (systolic and diastolic blood pressure and heart rate), number of HHFs in the previous year, number of HHFs in the previous 3 years, comorbidities (coronary artery disease, myocardial infarction, hypertension, dyslipidemia, diabetes mellitus, chronic kidney disease, dialysis, stroke, chronic obstructive pulmonary disease, peripheral arterial disease, and liver cirrhosis), medications used during the index admission (angiotensin-converting enzyme inhibitors [ACEIs] or angiotensin receptor blockers [ARBs], beta blockers, and 11 others), laboratory test results (serum creatinine levels and 15 others), echocardiography results, in-hospital events, and heart failure medications taken within 3 months of discharge (Table 1 and Table 2). The echocardiographic parameters of interest were the LVEF, left ventricular end-diastolic diameter, left ventricular end-systolic diameter, left atrium diameter, and mitral regurgitation severity. The in-hospital covariates during the index admission were hospital stay (in days), intensive care unit (ICU) stay (in days), episodes of shock (use of inotropic agents, intra-aortic balloon pumps, or extracorporeal membrane oxygenation), intubation, episodes of acute coronary syndrome, and percutaneous coronary interventions. The heart failure medications of interest were beta blockers, ivabradine, digoxin, ACEIs/ARBs, angiotensin receptor–neprilysin inhibitors (ARNIs), mineralocorticoid receptor antagonists (MRAs), and loop diuretics.

### 2.4. Outcome Definitions

The primary outcome was a composite of cardiovascular death or HHF during follow-up. The secondary outcomes were cardiovascular death, HHF, and all-cause mortality. HHF was defined as unscheduled hospitalization during which the patient required at least one treatment, which may have included diuretics, nitrites, or inotropic agents. The patients’ dates, places, and causes of death were linked to the Taiwan Death Registry database. The definition of cardiovascular death encompassed death due to acute myocardial infarction; sudden cardiac death; and death due to heart failure, stroke, cardiovascular procedures, cardiovascular hemorrhage, or other cardiovascular causes [18]. The follow-up period was defined as the period from the date of the index hospitalization to the date of death, outcome occurrence, or loss to follow-up or 31 December 2020, whichever occurred first.

### 2.5. Statistical Analysis

We categorized each patient into one of six ordinal groups on the basis of change in their heart rate from discharge to the 3-month screening period (decrease of ≥30 bpm, decrease of 20–29 bpm, decrease of 10–19 bpm, decrease of 0–9 bpm, increase of 1–10 bpm, and increase of >10 bpm). The associations among the baseline characteristics of the patients in the groups were tested using the Cochran–Armitage test for categorical variables, the general linear model for continuous variables, and the Jonckheere–Terpstra test for obviously skewed data (e.g., B-type natriuretic peptide levels). The association between the changes in the patients’ heart rates and their risk of outcomes was assessed using a Cox proportional-hazards model. The linear trend across the ordinal groups on the risk of outcomes was tested. In addition, we obtained the hazard ratios and corresponding confidence intervals using the ≥30-beats-per-minute decrease group as the reference group. We adjusted for all the covariates listed in Table 1 and Table 2 except the follow-up duration, including baseline heart rate and heart failure medications taken within 3 months of discharge, in the multivariable model. 

Since the cut-off values used to group the patients were subjective and arbitrary, we explored the possibility of a nonlinear relationship between changes in heart rate and risk of outcomes by treating heart rate reduction as a flexible restricted cubic spline. The locations of knots were set to the 5th, 35th, 65th, and 95th percentiles. We adjusted for the covariates in the restricted cubic spline model. Since our data set had some missing values, the Cox models (including the restricted cubic spline model) were calculated using the complete data after single expectation–maximization imputation. R (version 4.0.4, R Project for Statistical Computing) and the “rms” package (version 5.1 to 3.1) were used to generate the restricted cubic spline model. SAS (version 9.4, SAS Institute) was used for other statistical analyses. A two-sided *p*-value <0.05 was considered statistically significant.

## 3. Results

### 3.1. Patient Characteristics and Baseline Demographics

A total of 5236 patients were eligible in our analysis. The mean (± standard deviation) age was 63.0 ± 15.5 years, and nearly 70% of the patients were male. Of note, 15.9% of the subjects had been admitted for heart failure in the previous year. The most prevalent comorbidity was hypertension (67.4%), followed by coronary artery disease (57.3%), diabetes (48.2%), dyslipidemia (42.3%), and chronic kidney disease (39.7%). The most common medications prescribed for heart failure during the index admission were ACEIs/ARBs (86.3%), loop diuretics (84.1%), and beta-blockers (81.7%). The mean baseline LVEF was 30.2 ± 7.2%, and about one-thirds (33.6%) of the patients had moderate or severe mitral regurgitation. The mean hospital days was 13.1 ± 12.7 days, and the mean ICU duration was 1.8 ± 3.7 days. During the 3-month screening period after discharge, the most common medications prescribed for heart failure were ACEIs/ARBs (69.2%), beta-blockers (66.4%), and loop diuretics (65%). Of note, the average follow-up duration was 49.0 ± 38.1 months.

The results of patients’ characteristics and baseline demographics are listed in Table 1 and Table 2. More than half of the patients had diagnosed coronary artery disease (51.9% to 61.2%) and hypertension (63.4% to 69.2%), and 39.8% to 50.6% of the patients had diabetes mellitus. Most (74.1% to 86.1%) had no records of previous HHFs in the 3 years preceding the index admission. Most of the patients received standard treatments, including ACEIs/ARBs (83.8% to 90.1%) and beta blockers (75.0% to 86.8%), during the index hospitalization. Most (80.7% to 90.9%) of the patients were prescribed loop diuretics. The mean LVEFs of the different groups ranged from 29.1 ± 7.7% to 30.6 ± 7.0%. The patients’ hospital stay ranged from 11.9 ± 10.9 to 15.1 ± 11.6 days, of which the ICU constituted 1.2 ± 3.0 to 3.0 ± 4.6 days. In both cases, the most days were spent in the ≥30-bpm decrease group. Some of the patients experienced episodes of shock (12.0% to 23.2%) or respiratory failure (1.2% to 4.3%) during the index hospitalization. Heart failure medication coverage at the 3-month screening period was lower than that at admission (56.8–76.3% vs. 78.1–86.8% for beta blockers, and 66.4–75.7% vs. 87.6–90.1% for ACEIs/ARBs).

### 3.2. Changes in Heart Rate by the 3-Month Screening Period and Long-Term Outcomes

The primary outcome was the composite of HHF and cardiovascular death. The secondary outcomes were all-cause death, cardiovascular death, and HHF. The occurrences of the outcomes in each of the heart rate reduction groups are illustrated in Supplemental Table S1. According to the unadjusted Model 1, the occurrences of composite events increased significantly from the 10- to 19-bpm decrease group to the >10-bpm increase group (*p* for linear trend < 0.001, Model 1 in Table 3). The HHF also exhibited benefits among the patients’ whose heart rates decreased by ≥20 bpm (*p* for linear trend < 0.001). With adjustment for all the covariates except heart failure medications taken within 3 months of discharge, the model revealed significant dose–response relationships between heart rate reduction and the four outcomes of interest. The results indicate that smaller decreases in heart rates from discharge to 3-month screening period were associated with less favorable prognoses (a higher risk of all outcomes; *p* for linear trend < 0.05, Model 2 in Table 3). The results remained unchanged when we adjusted for heart failure medications taken within 3 months of discharge (*p* for linear trend < 0.05, Model 3 in Table 3).

**Figure 2:**
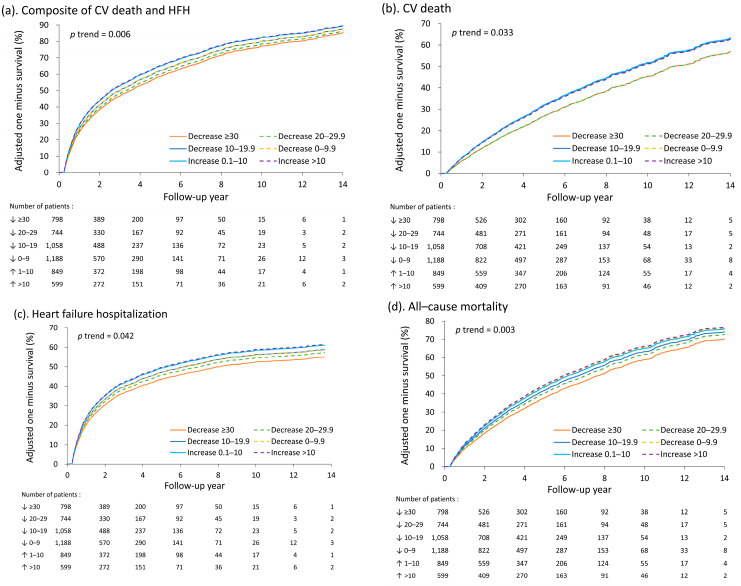
Adjusted (fitted) one minus survival for the composite outcome of cardiovascular death and HHF (**a**), cardiovascular death (**b**), HHF (**c**), and all-cause mortality (**d**) among patients with different changes in heart rate from discharge to 3-month screening period. HHF, hospitalization for heart failure; CV, cardiovascular.

**Figure 3:**
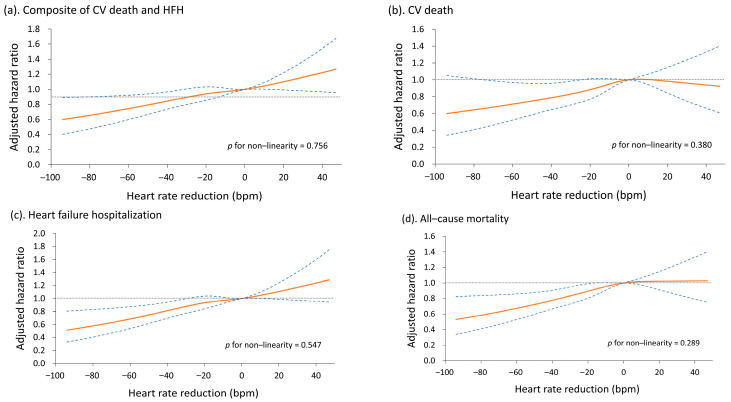
Relationship between changes in heart rate from discharge to 3-month screening period and risk of the composite outcome of cardiovascular death and HHF (**a**), cardiovascular death (**b**), HHF (**c**), and all-cause mortality (**d**) in patients with heart failure with reduced ejection fraction. HHF, hospitalization for heart failure; CV, cardiovascular.

The adjusted (fitted) survival rates of the patients are illustrated in Figure 2A–D. The possibility of a nonlinear relationship between heart rate reduction and risk of outcomes was further explored using the restricted cubic spline model. The results indicate that the relationship between heart rate reduction and risk of outcomes was linear *p* for nonlinearity > 0.2, Figure 3A–D). We also evaluated the association between heart rate at the 3-month screening period and risk of outcomes (Supplemental Tables S2–S4). Unsurprisingly, a higher heart rate was significantly associated with less favorable outcomes.

## 4. Discussion

We analyzed the long-term outcomes of patients with heart failure requiring hospitalization whose heart rates changed by various degrees during the study period. The results of this study indicate that optimal heart rate control can help patients with HFrEF avoid cardiovascular death, HHF, and all-cause mortality in the long term.

Our study demonstrated that heart rate reduction strategies may influence the long-term outcomes of patients with HFrEF. The patients whose heart rates decreased by ≥20 bpm after discharge (relative to their baseline heart rate at admission) had significantly more favorable prognoses. The results were consistent after we adjusted for the patients’ baseline characteristics and heart failure medications. Heart rate has often served as a monitoring target or predictive factor in studies on heart failure treatment [12]. A higher heart rate may indicate a more unstable condition. Analyses of data from European registries have revealed that patients have elevated heart rates when experiencing acute heart failure requiring admission [19,20]. However, the prognostic value of heart rate in acute heart failure remains controversial. One trial that enrolled patients hospitalized for acute heart failure identified baseline heart rate as a predictive factor for short-term adverse events [21]; however, Bertomeu-Gonzalez et al. observed that higher sinus rhythm heart rates at admission were not significantly associated with mortality [22]. Studies on the results of the Efficacy of Vasopressin Antagonism in Heart Failure Outcome Study with Tolvaptan (EVEREST) trial revealed that heart rate at admission is not correlated with long-term all-cause mortality in patients with HFrEF in sinus rhythm [20,23]. A higher baseline heart rate may be an indicator of sympathetic overactivity, greater oxygen consumption, a lower myocardial coronary perfusion time, and endothelial inflammation [7,24]. Nevertheless, previous literature has suggested that baseline heart rate alone is an insufficient prognostic indicator for patients with heart failure. 

Kurgansky et al. enrolled 51,194 patients with HFrEF with an LVEF of ≤35% in sinus rhythm from the US Veterans Affairs healthcare system. They discovered that a higher heart rate, both at the time of diagnosis and during follow-up, was strongly associated with an increased risk of adverse outcomes, [25] independent of the use of beta blockers. However, the results of our study indicate that the change between follow-up and baseline heart rate is more important. Our study differed in some respects from the large cohort study of Kurgansky et al. First, we enrolled patients hospitalized for HFrEF; therefore, the cardiovascular symptoms experienced by the patients may have been more severe. The higher MRA and loop diuretic use rates in our study also indicated the severity of patients with decompensated HFrEF. In addition, we enrolled patients with basal heart rates of ≥70 bpm, and excluded those with atrial fibrillation or atrial flutter. Another study conducted by Kotecha et al. revealed that using ß-blockers reduces mortality in patients of sinus rhythm with heart failure, irrespective of resting heart rate. Patients with heart rate <70 bpm were also enrolled in this meta-analysis [26]. Similarly to Kurgansky’s research, a higher resting heart rate at baseline and during follow-up increases mortality; however, patients without beta-blocker treatment experienced higher cardiac events. Proper treatment of heart rate for patients with heart failure of sinus rhythm could be beneficial. Our study also directed to the necessity of heart rate control. The beta-blocker usage rate at 3 months was highest in the ≥30-beats-per-minute decrease group, revealing a better long-term prognosis. However, the mean baseline heart rate of the ≥30-beats-per-minute decrease group was significantly higher than the mean across all the groups (113.9 bpm, *p* < 0.001). This group had more favorable long-term prognoses, including lower rates of mortality and HHF. The results suggest that the heart rate decreases in the 3 months after they began treatment was more important than the baseline heart rate of a patient with HFrEF. The benefits of heart rate reduction remained significant, even after adjustment for baseline heart rate, age, ejection fraction, heart failure medications, and other covariates. 

Heart rate control has been used as a treatment modality for heart failure for decades. As the standard treatment, beta blockers lower a patient’s heart rate, improve their sympathetic tone, reduce myocardial oxygen consumption, and control arrhythmia, resulting in more favorable clinical prognoses [13]. The beneficial effects of beta blockers strongly depend on their heart rate-reducing properties [27,28]. Ivabradine can be used to further reduce the heart rates of patients with HFrEF with sinus rhythm heart rates over 70 bpm, and help such patients achieve more favorable clinical outcomes, including a lower risk of mortality, especially when a patient’s heart rate can be reduced by ≥15 bpm [12,29]. One post hoc analysis of the EVEREST trial revealed that heart rates of ≥70 bpm after discharge are associated with an increased risk of mortality [23]. Nevertheless, heart rate reduction targets have rarely been discussed in the literature. Using our unadjusted models, we determined that a heart rate reduction of ≥20 bpm has significant benefits in terms of preventing the composite outcome of HHF and cardiovascular death. After adjustment for all covariates, the benefits remained significant. The overall mortality rate of the patients whose heart rates decreased by ≥30 bpm was significantly lower than that of the patients whose heart rates decreased by <10 bpm.

Compared with the patients enrolled in a previous study of registry data, [30] the patients in our study received more guideline-directed treatments during the index admission period, including beta blockers (78.1–86.8%), ACEIs/ARBs (83.8–90.1%), and MRAs (34.6–48.4%). During the follow-up period, the medication coverage rates (for beta blockers, ACEIs/ARBs, and MRAs) decreased. ACEIs/ARBs or MRAs may have been discontinued in our study because of hypotension or impaired renal function, since patients were with poor LV systolic function (EF 29.1–30.6%) or impaired renal function (Cr 2.0–2.2 mg/dL, eGFR 58–63). Beta blockers can have negative inotropic effects on cardiovascular hemodynamics, which causes many physicians to hesitate to prescribe or increase the dosage of such medications. Some physicians may change their patients’ prescriptions from beta blockers to other agents or discontinue beta blockers because their patients are intolerant to such medications. Finally, the ≥30-bpm decrease group had the fewest long-term cardiac events, but had the lowest mean LVEF (29.1 ± 7.7%, *p* < 0.001), highest mean initial heart rate (113.9 ± 18.0 bpm, *p* < 0.001), longest mean hospital and ICU stays, and highest incidence of shock events during the index admission period. These patients also had higher coverage rates of guideline-directed medications, including ACEIs/ARBs, beta blockers, ivabradine, and MRAs, during the follow-up period. The better guideline-directed medications coverage rate may be another reason for why this group achieved more favorable outcomes than did the other groups. However, after adjustment for all the covariates, including heart failure medications, greater heart rate reductions were still significantly associated with more favorable outcomes, including lower rates of overall mortality, and a lower incidence in the composite outcome of HHF and cardiovascular death. Our results are similar to those of a study by Hamill et al. that indicated that time-updated heart rates are more strongly related with cardiovascular outcomes than are baseline heart rates [31]. In the present study, only 20% of the patients (1062 of the 5236) had heart rates of <70 bpm at the 3-month screening period, indicating that heart rate management is often overlooked in the treatment of patients with chronic stable heart failure. Our study highlights the need to draw attention to this problem in the medical community, and to encourage early adoption of heart rate-lowering treatment strategies.

## 5. Limitations

Although this study provides key insights into the long-term clinical outcomes of heart rate control in patients with heart failure after hospitalization, it has some limitations. Firstly, because of the retrospective nature of this study, the different heart rate groups may have had inherent differences. The retrospective design also limited our ability to enroll patients randomly, and may have caused selection bias. Patients’ underlying conditions could have also altered the heart rate, including infection, inflammation, bleeding, or sepsis. Therefore, in our analysis, we adjusted for all the available covariates that may have been related to the outcomes. Secondly, heart rate was a key parameter in this study; however, data related to daily variations in the patients’ heart rates during follow-up were not collected. Heart rate from the in-hospital Holter devices would perhaps have been more reflective of the actual state. However, patients admitted with decompensated HFrEF seldom received Holter for heart rate recording in daily practice. This is also the limitation of the real-world retrospective analysis. Furthermore, the heart rate at admission was the condition before adequate and proper treatment. We recorded the heart rate upon admission as the baseline to compare; however, it could have been overestimated. Thirdly, undertaking physical activity and rehabilitation programs after acute exacerbations of heart failure may strongly affect a patient’s prognosis; however, information on the patients’ daily physical activity habits or rehabilitation statuses were unavailable in our database. Furthermore, this study only included patients in sinus rhythm; therefore, the effect of heart rate control on patients with atrial fibrillation still warrants further investigation. Finally, medication noncompliance may have occurred, and the information we obtained on the drugs prescribed to the patients may not have reflected the patients’ actual use of the drugs.

## 6. Conclusions

In this study, greater reductions in heart rate from discharge until the 3-month screening period were associated with a lower incidence of cardiovascular death, HHF, and all-cause mortality among patients discharged after hospitalization for decompensated HFrEF. Researchers should comprehensively evaluate guideline-directed therapies to determine which is most effective in helping patients achieve a target heart rate reduction and, in turn, more favorable long-term prognoses.

## Figures and Tables

**Table 1:** Baseline characteristics, comorbidities, laboratory data, and echocardiography results of patients grouped by changes in heart rate from the admission day to the 90th day after discharge.

Variable	*n*	Decrease ≥30 (*n* = 798)	Decrease 20–29 (*n* = 744)	Decrease 10–19 (*n* = 1058)	Decrease <10 (*n* = 1188)	Increase 1–10 (*n* = 849)	Increase >10 (*n* = 599)	*p* Trend
Age, year	5236	62.9 ± 16.7	63.6 ± 15.7	63.5 ± 15.2	63.2 ± 14.9	63.1 ± 15.1	61.0 ± 15.4	0.020
Male	5236	553 (69.3)	521 (70.0)	720 (68.1)	804 (67.7)	594 (70.0)	442 (73.8)	0.215
Smoking	5236	315 (39.5)	269 (36.2)	381 (36.0)	428 (36.0)	310 (36.5)	238 (39.7)	0.919
BMI, kg/m^2^	4920	24.9 ± 5.1	24.8 ± 4.6	25.0 ± 4.8	25.1 ± 4.7	25.1 ± 5.1	25.2 ± 5.1	0.092
Baseline vital sign								
SBP, mmHg	5236	134.9 ± 28.2	134.9 ± 27.2	134.6 ± 27.0	132.9 ± 24.8	129.3 ± 24.6	129.0 ± 23.2	<0.001
DBP, mmHg	5235	84.0 ± 20.1	81.7 ± 17.6	80.7 ± 18.4	78.7 ± 16.0	76.9 ± 16.0	77.9 ± 15.4	<0.001
Heart rate, beat/minute	5236	113.9 ± 18.0	98.3 ± 11.2	92.3 ± 12.1	87.0 ± 10.9	84.3 ± 10.2	81.6 ± 9.2	<0.001
HF admission in the previous year	5236	93 (11.7)	112 (15.1)	159 (15.0)	196 (16.5)	148 (17.4)	123 (20.5)	<0.001
No. of HF admission in the previous 3 years	5236							<0.001
0		687 (86.1)	613 (82.4)	866 (81.9)	933 (78.5)	659 (77.6)	444 (74.1)	
1		89 (11.2)	106 (14.2)	156 (14.7)	200 (16.8)	144 (17.0)	122 (20.4)	
≥2		22 (2.8)	25 (3.4)	36 (3.4)	55 (4.6)	46 (5.4)	33 (5.5)	
Comorbidity								
Coronary artery disease	5236	414 (51.9)	408 (54.8)	621 (58.7)	727 (61.2)	482 (56.8)	348 (58.1)	0.005
Myocardial infarction	5236	62 (7.8)	82 (11.0)	118 (11.2)	166 (14.0)	109 (12.8)	71 (11.9)	0.001
Hypertension	5236	506 (63.4)	499 (67.1)	732 (69.2)	817 (68.8)	584 (68.8)	393 (65.6)	0.170
Dyslipidemia	5236	299 (37.5)	310 (41.7)	443 (41.9)	539 (45.4)	385 (45.3)	239 (39.9)	0.024
Diabetes mellitus	5236	318 (39.8)	360 (48.4)	529 (50.0)	590 (49.7)	430 (50.6)	298 (49.7)	<0.001
Chronic kidney disease	5236	313 (39.2)	316 (42.5)	416 (39.3)	495 (41.7)	313 (36.9)	227 (37.9)	0.202
Dialysis	5236	48 (6.0)	76 (10.2)	109 (10.3)	118 (9.9)	88 (10.4)	61 (10.2)	0.015
Stroke	5236	52 (6.5)	62 (8.3)	79 (7.5)	103 (8.7)	62 (7.3)	46 (7.7)	0.547
Chronic obstructive Pulmonary disease	5236	139 (17.4)	142 (19.1)	160 (15.1)	205 (17.3)	167 (19.7)	108 (18.0)	0.475
Peripheral arterial disease	5236	55 (6.9)	63 (8.5)	103 (9.7)	101 (8.5)	87 (10.2)	68 (11.4)	0.005
Liver cirrhosis	5236	24 (3.0)	19 (2.6)	32 (3.0)	29 (2.4)	38 (4.5)	26 (4.3)	0.041
Laboratory data								
BNP, pg/mL	2884	1230 (599, 2239)	1168 (565, 2290)	1240 (612, 2449)	1100 (514, 2190)	1155 (509, 2580)	1166 (500, 2239)	0.382
BUN, mg/dL	5014	29.6 ± 20.9	31.2 ± 21.9	30.8 ± 22.8	31.0 ± 23.5	30.2 ± 21.7	30.1 ± 22.9	0.943
Creatinine, mg/dL	5219	2.0 ± 2.1	2.2 ± 2.4	2.2 ± 2.5	2.2 ± 2.7	2.2 ± 2.5	2.2 ± 2.6	0.364
eGFR, mL/min/1.73 m^2^	5219	58.0 ± 32.7	57.9 ± 35.1	59.3 ± 34.9	60.7 ± 36.4	61.8 ± 36.4	63.3 ± 36.4	<0.001
Sodium (Na), mEq/L	5197	137.8 ± 4.6	137.6 ± 4.5	137.9 ± 4.4	137.9 ± 4.3	138.1 ± 4.0	138.4 ± 3.9	0.001
Potassium (K), mEq/L	5204	3.9 ± 0.7	4.0 ± 0.7	4.0 ± 0.6	4.0 ± 0.6	4.0 ± 0.6	4.0 ± 0.6	0.045
Uric acid, mg/dL	3275	8.0 ± 2.8	7.8 ± 2.5	7.7 ± 2.4	7.5 ± 2.5	7.7 ± 2.5	7.7 ± 2.8	0.028
AST, U/L	4014	34 (24, 62)	31 (22, 49)	29 (22, 45)	30 (22, 46)	28 (21, 42)	29 (21, 44)	<0.001
ALT, U/L	4833	27 (17, 54)	25 (16, 45)	24 (15, 42)	25 (16, 44)	24 (16, 40)	22 (14, 38)	<0.001
LDL-C, mg/dL	4131	82.9 ± 47.8	83.8 ± 48.1	85.7 ± 49.7	86.6 ± 50.1	86.0 ± 49.2	88.1 ± 48.8	0.056
Total cholesterol, mg/dL	4275	167.7 ± 44.5	168.6 ± 46.8	167.8 ± 45.6	170.3 ± 45.3	166.6 ± 45.9	167.8 ± 44.9	0.859
Hemoglobin, g/dL	5228	12.7 ± 2.6	12.5 ± 2.6	12.5 ± 2.5	12.4 ± 2.5	12.4 ± 2.5	12.6 ± 2.4	0.613
Total bilirubin, mg/dL	3315	1.1 ± 0.9	1.0 ± 0.8	0.9 ± 0.7	0.9 ± 0.7	0.9 ± 0.7	0.9 ± 0.6	<0.001
Albumin, mg/dL	3734	3.5 ± 0.6	3.5 ± 0.6	3.5 ± 0.6	3.5 ± 0.6	3.5 ± 0.6	3.5 ± 0.5	0.023
Platelet, count × 10^3^	5225	233.2 ± 86.1	224.3 ± 84.3	227.6 ± 82.4	218.2 ± 75.1	217.5 ± 82.8	220.9 ± 76.5	<0.001
WBC, count × 10^3^	5228	10.8 ± 4.7	9.3 ± 3.8	9.2 ± 3.8	8.6 ± 3.5	8.5 ± 3.5	8.5 ± 3.2	<0.001
Echocardiography result								
LVEF, %	5236	29.1 ± 7.7	29.8 ± 7.5	30.2 ± 7.3	30.5 ± 7.0	30.5 ± 6.9	30.6 ± 7.0	<0.001
LVEDD, mm	5233	58.7 ± 9.1	59.1 ± 8.6	59.4 ± 8.9	59.0 ± 8.6	59.5 ± 8.4	59.6 ± 8.4	0.047
LVESD, mm	5231	49.8 ± 9.4	49.8 ± 8.8	49.9 ± 9.8	49.6 ± 8.7	49.9 ± 8.8	50.2 ± 8.6	0.530
LA diameter, mm	5194	42.5 ± 8.2	42.5 ± 7.4	42.8 ± 7.7	42.4 ± 7.7	42.3 ± 7.6	42.5 ± 7.9	0.719
MR severity	5236							0.133
Severe		47 (5.9)	44 (5.9)	92 (8.7)	92 (7.7)	81 (9.5)	49 (8.2)	
Moderate		214 (26.8)	201 (27.0)	266 (25.1)	307 (25.8)	221 (26.0)	146 (24.4)	
Mild		440 (55.1)	411 (55.2)	590 (55.8)	660 (55.6)	457 (53.8)	348 (58.1)	
Trivial/None		89 (11.2)	79 (10.6)	96 (9.1)	122 (10.3)	82 (9.7)	51 (8.5)	
Follow up duration, month	5236	46.4 ± 36.4	47.4 ± 38.4	48.4 ± 36.8	49.8 ± 38.2	49.7 ± 39.1	52.6 ± 40.3	0.001

Data are presented as frequencies (percentages) or means ± standard deviations. Abbreviations: BMI, body mass index; SBP, systolic blood pressure; DBP, diastolic blood pressure; HF, heart failure; BNP, B-type natriuretic peptide; BUN, blood urea nitrogen; eGFR, estimated glomerular filtration rate; AST, aspartate aminotransferase; ALT, alanine amino transferase; LDL-C, low-density lipoprotein cholesterol; WBC, white blood cell; LVEF, left ventricular ejection fraction; LVEDD, left ventricular end-diastolic dimension; LVESD, left ventricular end-systolic diameter; LA, left atrium; MR, mitral regurgitation; ICU, intensive care unit; PCI, percutaneous coronary intervention.

**Table 2:** Medications and in-hospital events of patients grouped by changes in heart rate from the admission day to the 90th day after discharge.

Variable	*n*	Decrease ≥30 (*n* = 798)	Decrease 20–29 (*n* = 744)	Decrease 10–19 (*n* = 1058)	Decrease <10 (*n* = 1188)	Increase 1–10 (*n* = 849)	Increase >10 (*n* = 599)	*p* Trend
Medication for heart failure during the index admission								
ARNI	5236	18 (2.3)	19 (2.6)	27 (2.6)	23 (1.9)	27 (3.2)	13 (2.2)	0.811
ACEI/ARB	5236	719 (90.1)	651 (87.5)	916 (86.6)	995 (83.8)	714 (84.1)	525 (87.6)	0.004
Beta-blocker	5236	693 (86.8)	648 (87.1)	879 (83.1)	953 (80.2)	637 (75.0)	468 (78.1)	<0.001
Ivabradine	5236	146 (18.3)	71 (9.5)	86 (8.1)	59 (5.0)	36 (4.2)	25 (4.2)	<0.001
MRAs	5236	386 (48.4)	317 (42.6)	462 (43.7)	459 (38.6)	308 (36.3)	207 (34.6)	<0.001
Loop diuretics	5236	725 (90.9)	648 (87.1)	895 (84.6)	965 (81.2)	685 (80.7)	488 (81.5)	<0.001
Digoxin	5236	145 (18.2)	100 (13.4)	147 (13.9)	177 (14.9)	121 (14.3)	94 (15.7)	0.325
Other medication during the index admission								
DHP-CCB	5236	302 (37.8)	293 (39.4)	437 (41.3)	433 (36.4)	283 (33.3)	211 (35.2)	0.010
Amiodarone	5236	70 (8.8)	42 (5.6)	64 (6.0)	66 (5.6)	38 (4.5)	26 (4.3)	<0.001
Oral hypoglycemic agents	5236	266 (33.3)	307 (41.3)	432 (40.8)	489 (41.2)	343 (40.4)	237 (39.6)	0.032
Insulin	5236	266 (33.3)	238 (32.0)	352 (33.3)	346 (29.1)	240 (28.3)	186 (31.1)	0.029
Statin	5236	363 (45.5)	356 (47.8)	518 (49.0)	595 (50.1)	371 (43.7)	274 (45.7)	0.579
Aspirin	5236	538 (67.4)	523 (70.3)	760 (71.8)	873 (73.5)	596 (70.2)	418 (69.8)	0.239
P2Y12	5236	423 (53.0)	400 (53.8)	586 (55.4)	623 (52.4)	434 (51.1)	303 (50.6)	0.133
In-hospital event								
Hospital days	5236	15.1 ± 11.6	14.2 ± 12.2	13.8 ± 14.5	11.8 ± 10.4	12.1 ± 15.4	11.9 ± 10.9	<0.001
ICU days	5236	3.0 ± 4.6	2.0 ± 3.8	2.0 ± 4.1	1.4 ± 3.4	1.1 ± 2.9	1.2 ± 3.0	<0.001
Shock	5236	185 (23.2)	111 (14.9)	169 (16.0)	153 (12.9)	102 (12.0)	91 (15.2)	<0.001
Intubation	5236	34 (4.3)	22 (3.0)	38 (3.6)	24 (2.0)	10 (1.2)	10 (1.7)	<0.001
Acute coronary syndrome	5236	217 (27.2)	166 (22.3)	232 (21.9)	229 (19.3)	140 (16.5)	102 (17.0)	<0.001
PCI	5236	133 (16.7)	142 (19.1)	184 (17.4)	196 (16.5)	148 (17.4)	92 (15.4)	0.343
Medication for heart failure within 3 months after discharge								
Beta-blocker	5236	609 (76.3)	559 (75.1)	706 (66.7)	748 (63.0)	515 (60.7)	340 (56.8)	<0.001
Ivabradine	5236	113 (14.2)	58 (7.8)	61 (5.8)	51 (4.3)	33 (3.9)	23 (3.8)	<0.001
Digoxin	5236	109 (13.7)	70 (9.4)	105 (9.9)	121 (10.2)	82 (9.7)	78 (13.0)	0.535
ACEi/ARB	5236	604 (75.7)	539 (72.4)	715 (67.6)	790 (66.5)	564 (66.4)	411 (68.6)	<0.001
ARNI	5236	16 (2.0)	14 (1.9)	24 (2.3)	16 (1.3)	23 (2.7)	11 (1.8)	0.876
MRAs	5236	321 (40.2)	260 (34.9)	366 (34.6)	363 (30.6)	234 (27.6)	163 (27.2)	<0.001
Loop diuretics	5236	554 (69.4)	520 (69.9)	687 (64.9)	756 (63.6)	523 (61.6)	366 (61.1)	<0.001

Data are presented as frequencies (percentages) or means ± standard deviations. Abbreviations: ACEIs, angiotensin-converting enzyme inhibitors; ARBs, angiotensin receptor blockers; MRAs, mineralocorticoid receptor antagonists; P2Y_12_, purinergic receptor P2Y, G-protein coupled, 12.

**Table 3:** Outcomes of patients grouped by changes in heart rate.

Outcome	Decrease ≥30 (*n* = 798)	Decrease 20–29 (*n* = 744)	Decrease 10–19 (*n* = 1058)	Decrease <10 (*n* = 1188)	Increase 1–10 (*n* = 849)	Increase >10 (*n* = 599)	*p* Trend
Composite of heart failure hospitalization and cardiovascular death							
Model 1	Ref	1.08 (0.94–1.24)	1.15 (1.01–1.30) *	1.15 (1.02–1.30) *	1.25 (1.10–1.42)*	1.24 (1.07–1.42) *	<0.001
Model 2	Ref	1.05 (0.91–1.22)	1.12 (0.97–1.30)	1.14 (0.97–1.32)	1.23 (1.04–1.45)*	1.25 (1.05–1.50) *	0.003
Model 3	Ref	1.05 (0.91–1.22)	1.12 (0.96–1.29)	1.13 (0.97–1.31)	1.22 (1.03–1.44)*	1.24 (1.03–1.48) *	0.006
Cardiovascular death							
Model 1	Ref	1.03 (0.84–1.26)	1.19 (0.99–1.42)	1.09 (0.92–1.31)	1.12 (0.93–1.36)	1.07 (0.87–1.32)	0.404
Model 2	Ref	1.01 (0.81–1.26)	1.30 (1.05–1.61) *	1.29 (1.03–1.62) *	1.31 (1.03–1.67) *	1.33 (1.02–1.73) *	0.012
Model 3	Ref	1.01 (0.81–1.25)	1.27 (1.02–1.57) *	1.25 (0.998–1.57)	1.27 (0.998–1.63)	1.27 (0.97–1.66)	0.033
Heart failure hospitalization							
Model 1	Ref	1.08 (0.93–1.26)	1.16 (1.01–1.33) *	1.19 (1.04–1.36) *	1.27 (1.10–1.47) *	1.26 (1.08–1.47) *	<0.001
Model 2	Ref	1.07 (0.90–1.26)	1.12 (0.95–1.32)	1.13 (0.95–1.34)	1.20 (1.002–1.45) *	1.23 (1.003–1.50) *	0.027
Model 3	Ref	1.07 (0.91–1.27)	1.12 (0.95–1.32)	1.12 (0.94–1.33)	1.20 (0.99–1.44)	1.22 (0.99–1.49)	0.042
All-cause mortality							
Model 1	Ref	1.15 (0.98–1.33)	1.11 (0.96–1.27)	1.13 (0.98–1.30)	1.12 (0.97–1.30)	1.14 (0.97–1.33)	0.197
Model 2	Ref	1.12 (0.95–1.32)	1.19 (1.01–1.41)*	1.33 (1.11–1.58) *	1.29 (1.07–1.56) *	1.38 (1.12–1.69) *	0.001
Model 3	Ref	1.11 (0.94–1.31)	1.17 (0.99–1.39)	1.30 (1.09–1.55) *	1.26 (1.05–1.53) *	1.34 (1.09–1.64) *	0.003

* *p* <0.05; Model 1: unadjusted; Model 2: adjusted for all covariates (number of covariates = 54) listed in Table 1 and Table 2, except follow-up duration, heart failure medications during the admission, and heart failure medications within 3 months after discharge; Model 3: adjusted for all the covariates listed in Table 1 and Table 2 (number of covariates = 68), except follow-up duration.

## Data Availability

The data presented in this study are available on request from the corresponding author. The data are not publicly available due to ethical regulation of the database.

## Supplementary Materials

The following supporting information can be downloaded at: https://www.mdpi.com/article/10.3390/medicina59020348/s1, Table S1: Occurrence of outcomes among patients grouped by changes in heart rate; Table S2: Baseline characteristics of patients grouped by heart rate at the 90th day after discharge; Table S3: Occurrence of outcomes among patients grouped by heart rate; Table S4: Outcomes of patients grouped by heart rate at the 90th day after discharge
