# Supplementary material for: Optimal Heart Rate Control Improves Long-Term Prognosis of Decompensated Heart Failure with Reduced Ejection Fraction
Source: Medicina (Kaunas). 2023 Feb 12;59(2):348. doi: 10.3390/medicina59020348 (PMC9968049; doi:10.3390/medicina59020348)
Supplement: Supplementary file 1 [file medicina-59-00348-s001.zip › medicina-2132442-supplementary.pdf]

Supplemental Table S1. Occurrence of outcomes among patients grouped by changes in heart rate

Variable	Decrease ≥30 (*n* = 798)	Decrease 20-29 (*n* = 744)	Decrease 10-19 (*n* = 1,058)	Decrease <10 (*n* = 1,188)	Increase 1-10 (*n* = 849)	Increase >10 (*n* = 599)
Composite of heart failure hospitalization and cardiovascular death	414 (51.9)	397 (53.4)	609 (57.6)	689 (58.0)	510 (60.1)	367 (61.3)
Cardiovascular death	191 (23.9)	187 (25.1)	314 (29.7)	333 (28.0)	243 (28.6)	173 (28.9)
Heart failure admission	329 (41.2)	326 (43.8)	491 (46.4)	571 (48.1)	424 (49.9)	303 (50.6)
All-cause mortality	317 (39.7)	345 (46.4)	484 (45.7)	570 (48.0)	403 (47.5)	304 (50.8)

Data are presented as frequencies (percentages).

Supplemental Table S2. Baseline characteristics of patients grouped by heart rate at the 90th day after discharge

Variable	*N*	<60 (*n* = 271)	60-69 (*n* = 791)	70-79 (*n* = 1,326)	80-89 (*n* = 1,384)	90-99 (*n* = 839)	≥100 (*n* = 625)	*P* trend
Age, year	5,236	68.0 ± 15.5	66.7 ± 14.4	64.0 ± 15.2	62.6 ± 15.2	61.2 ± 14.9	57.5 ± 16.7	<0.001
Male	5,236	194 (71.6)	513 (64.9)	913 (68.9)	970 (70.1)	589 (70.2)	455 (72.8)	0.015
Smoking	5,236	98 (36.2)	267 (33.8)	462 (34.8)	525 (37.9)	323 (38.5)	266 (42.6)	<0.001
BMI, kg/m^2	4,920	24.5 ± 4.4	24.5 ± 4.5	25.1 ± 4.6	24.8 ± 4.9	25.3 ± 5.1	25.7 ± 5.6	<0.001
Baseline vital sign								
SBP, mmHg	5,236	130.0 ± 23.5	136.7 ± 27.7	133.2 ± 26.0	132.3 ± 25.9	131.1 ± 25.8	131.7 ± 25.1	0.374
DBP, mmHg	5,235	79.1 ± 17.6	79.7 ± 17.9	79.9 ± 17.5	79.2 ± 17.1	79.7 ± 17.4	82.8 ± 17.5	0.010
Heart rate, bpm	5,236	90.7 ± 17.7	90.1 ± 16.1	90.6 ± 16.1	92.5 ± 15.1	95.7 ± 15.6	98.2 ± 15.2	<0.001
HF admission in the previous year	5,236	38 (14.0)	102 (12.9)	213 (16.1)	210 (15.2)	154 (18.4)	114 (18.2)	0.002
No. of HF admission in the previous 3 years	5,236							0.023
0		223 (82.3)	662 (83.7)	1,064 (80.2)	1,119 (80.9)	643 (76.6)	491 (78.6)	
1		37 (13.7)	100 (12.6)	213 (16.1)	200 (14.5)	156 (18.6)	111 (17.8)	
≥2		11 (4.1)	29 (3.7)	49 (3.7)	65 (4.7)	40 (4.8)	23 (3.7)	
Comorbidity								
Coronary artery disease	5,236	150 (55.4)	489 (61.8)	794 (59.9)	792 (57.2)	484 (57.7)	291 (46.6)	<0.001
Myocardial infarction	5,236	32 (11.8)	90 (11.4)	175 (13.2)	159 (11.5)	101 (12.0)	51 (8.2)	0.065
Hypertension	5,236	191 (70.5)	559 (70.7)	913 (68.9)	934 (67.5)	547 (65.2)	387 (61.9)	<0.001
Dyslipidemia	5,236	111 (41.0)	359 (45.4)	568 (42.8)	605 (43.7)	363 (43.3)	209 (33.4)	0.002
Diabetes mellitus	5,236	93 (34.3)	363 (45.9)	649 (48.9)	703 (50.8)	451 (53.8)	266 (42.6)	0.019
Chronic kidney disease	5,236	98 (36.2)	319 (40.3)	542 (40.9)	565 (40.8)	328 (39.1)	228 (36.5)	0.389

Variable	*N*	<60 (*n* = 271)	60-69 (*n* = 791)	70-79 (*n* = 1,326)	80-89 (*n* = 1,384)	90-99 (*n* = 839)	≥100 (*n* = 625)	*P* trend
Dialysis	5,236	14 (5.2)	75 (9.5)	125 (9.4)	138 (10.0)	80 (9.5)	68 (10.9)	0.059
Stroke	5,236	20 (7.4)	68 (8.6)	95 (7.2)	119 (8.6)	59 (7.0)	43 (6.9)	0.446
Chronic obstructive pulmonary disease	5,236	50 (18.5)	129 (16.3)	219 (16.5)	254 (18.4)	157 (18.7)	112 (17.9)	0.275
Peripheral arterial disease	5,236	21 (7.7)	64 (8.1)	126 (9.5)	125 (9.0)	87 (10.4)	54 (8.6)	0.348
Liver cirrhosis	5,236	10 (3.7)	29 (3.7)	31 (2.3)	46 (3.3)	26 (3.1)	26 (4.2)	0.514
Medication for heart failure during the index admission								
ARNI	5,236	11 (4.1)	23 (2.9)	32 (2.4)	31 (2.2)	18 (2.1)	12 (1.9)	0.053
ACEI/ARB	5,236	239 (88.2)	687 (86.9)	1,156 (87.2)	1,176 (85.0)	718 (85.6)	544 (87.0)	0.343
Beta-blocker	5,236	229 (84.5)	707 (89.4)	1,112 (83.9)	1,117 (80.7)	656 (78.2)	457 (73.1)	<0.001
Ivabradine	5,236	33 (12.2)	82 (10.4)	102 (7.7)	97 (7.0)	68 (8.1)	41 (6.6)	0.002
MRAs	5,236	125 (46.1)	345 (43.6)	536 (40.4)	543 (39.2)	329 (39.2)	261 (41.8)	0.086
Loop diuretics	5,236	238 (87.8)	667 (84.3)	1,098 (82.8)	1,143 (82.6)	724 (86.3)	536 (85.8)	0.518
Digoxin	5,236	47 (17.3)	86 (10.9)	206 (15.5)	192 (13.9)	135 (16.1)	118 (18.9)	0.006
Other medication during the index admission								
DHP-CCB	5,236	99 (36.5)	310 (39.2)	503 (37.9)	518 (37.4)	319 (38.0)	210 (33.6)	0.141
Amiodarone	5,236	22 (8.1)	56 (7.1)	90 (6.8)	79 (5.7)	36 (4.3)	23 (3.7)	<0.001
Oral hypoglycemic agents	5,236	72 (26.6)	296 (37.4)	513 (38.7)	589 (42.6)	380 (45.3)	224 (35.8)	0.001
Insulin	5,236	59 (21.8)	223 (28.2)	395 (29.8)	460 (33.2)	300 (35.8)	191 (30.6)	<0.001
Statin	5,236	128 (47.2)	410 (51.8)	673 (50.8)	647 (46.7)	387 (46.1)	232 (37.1)	<0.001

Variable	*N*	<60 (*n* = 271)	60-69 (*n* = 791)	70-79 (*n* = 1,326)	80-89 (*n* = 1,384)	90-99 (*n* = 839)	≥100 (*n* = 625)	*P* trend
Aspirin	5,236	189 (69.7)	573 (72.4)	979 (73.8)	964 (69.7)	614 (73.2)	389 (62.2)	0.001
P2Y12	5,236	147 (54.2)	456 (57.6)	717 (54.1)	735 (53.1)	450 (53.6)	264 (42.2)	<0.001
Laboratory data								
BNP, pg/mL	2,884	1273 [719, 2427]	1177 [563, 2511]	1220 [533, 2400]	1211 [553, 2317]	1059 [536, 2020]	1147 [554, 2190]	0.244
BUN, mg/dL	5,014	30.8 ± 21.4	30.8 ± 21.3	30.3 ± 21.5	30.9 ± 23.0	30.5 ± 23.8	30.1 ± 22.8	0.702
Creatinine, mg/dL	5,219	1.9 ± 1.9	2.1 ± 2.2	2.1 ± 2.3	2.2 ± 2.5	2.2 ± 2.7	2.3 ± 2.9	0.016
eGFR, mL/min/1.73m^2	5,219	57.5 ± 30.8	56.4 ± 33.5	58.7 ± 34.0	60.1 ± 35.7	63.0 ± 37.4	64.9 ± 38.3	<0.001
Sodium (Na), mEq/L	5,197	138.4 ± 4.0	137.8 ± 4.5	138.1 ± 4.3	137.9 ± 4.2	137.6 ± 4.4	137.9 ± 4.3	0.055
Potassium (K), mEq/L	5,204	3.9 ± 0.6	4.0 ± 0.6	4.0 ± 0.6	4.0 ± 0.6	4.0 ± 0.6	4.0 ± 0.6	0.333
Uric acid, mg/dL	3,275	8.2 ± 2.9	7.7 ± 2.5	7.6 ± 2.6	7.6 ± 2.5	7.7 ± 2.6	7.9 ± 2.7	0.230
AST, U/L	4,014	32 [24, 48]	30 [22, 50]	29 [22, 47]	30 [21, 47]	30 [21, 47]	30 [22, 46]	0.579
ALT, U/L	4,833	25 [15, 44]	25 [16, 44]	25 [16, 41]	24 [16, 42]	24 [16, 45]	25 [16, 46]	0.961
LDL-C, mg/dL	4,131	79.2 ± 45.2	86.4 ± 49.0	84.3 ± 49.9	88.4 ± 49.5	80.1 ± 48.1	90.7 ± 48.7	0.053
Total cholesterol, mg/dL	4,275	167.1 ± 40.4	172.1 ± 45.0	166.9 ± 44.0	168.5 ± 48.1	165.1 ± 44.0	170.5 ± 47.5	0.895
Hemoglobin, g/dL	5,228	12.7 ± 2.5	12.3 ± 2.5	12.6 ± 2.5	12.4 ± 2.5	12.4 ± 2.6	12.8 ± 2.5	0.550
Total bilirubin, mg/dL	3,315	1.0 ± 0.8	0.9 ± 0.7	0.9 ± 0.7	0.9 ± 0.7	0.9 ± 0.8	1.0 ± 0.8	0.920
Albumin, mg/dL	3,734	3.5 ± 0.6	3.5 ± 0.6	3.5 ± 0.6	3.5 ± 0.6	3.5 ± 0.5	3.5 ± 0.5	0.108
Platelet, count × 10^3	5,225	210.0 ± 66.2	222.5 ± 85.0	217.7 ± 77.9	223.0 ± 79.8	229.4 ± 84.7	235.4 ± 85.5	<0.001
WBC, count × 10^3	5,228	9.4 ± 4.0	9.1 ± 3.9	9.1 ± 4.0	9.0 ± 3.7	9.2 ± 3.8	9.3 ± 3.6	0.879
Echocardiography result								
LVEF, %	5,236	29.6 ± 7.4	30.4 ± 7.5	30.3 ± 7.1	30.5 ± 7.0	29.9 ± 7.4	29.4 ± 7.4	0.355

Variable	*N*	<60 (*n* = 271)	60-69 (*n* = 791)	70-79 (*n* = 1,326)	80-89 (*n* = 1,384)	90-99 (*n* = 839)	≥100 (*n* = 625)	*P* trend
LVEDD, mm	5,233	60.1 ± 9.7	58.8 ± 8.5	59.1 ± 8.7	59.2 ± 8.8	59.2 ± 8.7	59.9 ± 8.2	0.980
LVESD, mm	5,231	50.5 ± 10.0	49.3 ± 8.9	49.7 ± 8.9	49.7 ± 9.4	49.8 ± 9.2	50.8 ± 8.4	0.419
LA diameter, mm	5,194	43.4 ± 7.6	42.3 ± 7.6	42.5 ± 7.6	42.2 ± 7.9	42.3 ± 7.5	43.4 ± 8.0	0.976
MR severity	5,236							0.013
Severe		14 (5.2)	51 (6.4)	90 (6.8)	112 (8.1)	79 (9.4)	59 (9.4)	
Moderate		69 (25.5)	196 (24.8)	355 (26.8)	345 (24.9)	233 (27.8)	157 (25.1)	
Mild		160 (59.0)	461 (58.3)	717 (54.1)	766 (55.3)	448 (53.4)	354 (56.6)	
Trivial/None		25 (9.2)	74 (9.4)	151 (11.4)	144 (10.4)	74 (8.8)	51 (8.2)	
In-hospital event								
Hospital days	5,236	13.2 ± 11.6	13.1 ± 11.7	12.9 ± 11.2	13.1 ± 13.8	13.7 ± 14.9	13.0 ± 11.9	0.826
ICU days	5,236	2.2 ± 4.7	1.8 ± 3.1	1.8 ± 3.8	1.7 ± 3.7	1.8 ± 3.7	1.4 ± 3.7	0.007
Shock	5,236	43 (15.9)	122 (15.4)	201 (15.2)	207 (15.0)	142 (16.9)	96 (15.4)	0.721
Intubation	5,236	8 (3.0)	18 (2.3)	42 (3.2)	32 (2.3)	21 (2.5)	17 (2.7)	0.801
Acute coronary syndrome	5,236	71 (26.2)	190 (24.0)	295 (22.2)	281 (20.3)	163 (19.4)	86 (13.8)	<0.001
PCI	5,236	46 (17.0)	141 (17.8)	244 (18.4)	248 (17.9)	136 (16.2)	80 (12.8)	0.017
Medication for heart failure within 3 months after discharge								
Beta-blocker	5,236	194 (71.6)	610 (77.1)	925 (69.8)	888 (64.2)	502 (59.8)	358 (57.3)	<0.001
Ivabradine	5,236	29 (10.7)	70 (8.8)	74 (5.6)	81 (5.9)	54 (6.4)	31 (5.0)	0.001
Digoxin	5,236	36 (13.3)	61 (7.7)	139 (10.5)	128 (9.2)	100 (11.9)	101 (16.2)	<0.001
ACEi/ARB	5,236	196 (72.3)	560 (70.8)	918 (69.2)	935 (67.6)	564 (67.2)	450 (72.0)	0.473

Variable	*N*	<60 (*n* = 271)	60-69 (*n* = 791)	70-79 (*n* = 1,326)	80-89 (*n* = 1,384)	90-99 (*n* = 839)	≥100 (*n* = 625)	*P* trend
ARNI	5,236	8 (3.0)	19 (2.4)	27 (2.0)	27 (2.0)	15 (1.8)	8 (1.3)	0.063
MRAs	5,236	101 (37.3)	279 (35.3)	439 (33.1)	419 (30.3)	263 (31.3)	206 (33.0)	0.045
Loop diuretics	5,236	196 (72.3)	513 (64.9)	828 (62.4)	870 (62.9)	574 (68.4)	425 (68.0)	0.355
Follow up duration, month	5,236	44.7 ± 33.8	46.8 ± 35.6	49.5 ± 38.8	50.1 ± 38.9	48.1 ± 38.3	51.1 ± 39.3	0.015

Abbreviations: BMI, body mass index; SBP, systolic blood pressure; DBP, diastolic blood pressure; HF, heart failure; ARNIs, angiotensin receptor–neprilysin inhibitors; ACEIs, angiotensin-converting enzyme inhibitors; ARBs, angiotensin receptor blockers; MRAs, mineralocorticoid receptor antagonists; $P2Y_{12}$, purinergic receptor P2Y, G-protein coupled, 12; BNP, B-type natriuretic peptide; BUN, blood urea nitrogen; eGFR, estimated glomerular filtration rate; AST, aspartate aminotransferase; ALT, alanine amino transferase; LDL-C, low-density lipoprotein cholesterol; WBC, white blood cell; LVEF, left ventricular ejection fraction; LVEDD, left ventricular end-diastolic dimension; LVESD, left ventricular end-systolic diameter; LA, left atrium; MR, mitral regurgitation; ICU, intensive care unit; PCI, percutaneous coronary intervention.

Data are presented as frequencies (percentages) or means ± standard deviations.

Supplemental Table S3. Occurrence of outcomes among patients grouped by heart rate

Outcome	<60 (*n* = 271)	60-69 (*n* = 791)	70-79 (*n* = 1,326)	80-89 (*n* = 1,384)	90-99 (*n* = 839)	≥100 (*n* = 625)
Composite of heart failure hospitalization and cardiovascular death	159 (58.7)	451 (57.0)	758 (57.2)	777 (56.1)	476 (56.7)	365 (58.4)
Cardiovascular death	83 (30.6)	211 (26.7)	359 (27.1)	396 (28.6)	233 (27.8)	159 (25.4)
Heart failure admission	126 (46.5)	364 (46.0)	615 (46.4)	631 (45.6)	395 (47.1)	313 (50.1)
All-cause mortality	137 (50.6)	354 (44.8)	601 (45.3)	650 (47.0)	396 (47.2)	285 (45.6)

Data are presented as frequencies (percentages).

Supplemental Table S4. Outcomes of patients grouped by heart rate at the 90th day after discharge

Variable	<60 (*n* = 271)	60-69 (*n* = 791)	70-79 (*n* = 1,326)	80-89 (*n* = 1,384)	90-99 (*n* = 839)	≥100 (*n* = 625)	*P* trend
Composite of heart failure hospitalization and cardiovascular death							
Model 1	Ref	0.93 (0.77–1.11)	0.93 (0.79–1.11)	0.91 (0.76–1.08)	0.94 (0.79–1.13)	1.01 (0.84–1.22)	0.584
Model 2	Ref	0.95 (0.79–1.14)	1.01 (0.85–1.21)	0.99 (0.83–1.18)	1.07 (0.89–1.29)	1.29 (1.06–1.56)*	<0.001
Model 3	Ref	0.96 (0.80–1.15)	1.02 (0.86–1.22)	1.00 (0.84–1.19)	1.07 (0.89–1.29)	1.29 (1.06–1.56)*	0.001
Cardiovascular death							
Model 1	Ref	0.83 (0.65–1.08)	0.80 (0.63–1.01)	0.84 (0.66–1.06)	0.85 (0.66–1.09)	0.73 (0.56–0.95)*	0.155
Model 2	Ref	0.94 (0.73–1.22)	0.99 (0.77–1.26)	1.07 (0.84–1.36)	1.18 (0.91–1.53)	1.14 (0.87–1.51)	0.013
Model 3	Ref	0.95 (0.73–1.23)	0.98 (0.77–1.26)	1.05 (0.83–1.35)	1.15 (0.89–1.50)	1.12 (0.85–1.47)	0.039
Heart failure admission							
Model 1	Ref	0.95 (0.77–1.16)	0.96 (0.80–1.17)	0.94 (0.77–1.13)	0.99 (0.81–1.22)	1.10 (0.90–1.35)	0.136
Model 2	Ref	0.95 (0.77–1.17)	1.02 (0.84–1.24)	1.00 (0.82–1.21)	1.08 (0.88–1.33)	1.35 (1.09–1.67)*	<0.001
Model 3	Ref	0.97 (0.79–1.19)	1.03 (0.85–1.25)	1.01 (0.83–1.22)	1.08 (0.88–1.33)	1.35 (1.09–1.67)*	0.001
All-cause mortality							
Model 1	Ref	0.85 (0.70–1.03)	0.81 (0.67–0.98)*	0.83 (0.69–1.00)	0.87 (0.72–1.06)	0.79 (0.65–0.97)*	0.282
Model 2	Ref	0.90 (0.74–1.10)	0.95 (0.79–1.15)	0.98 (0.81–1.19)	1.12 (0.92–1.37)	1.16 (0.94–1.44)	0.001
Model 3	Ref	0.91 (0.74–1.11)	0.95 (0.79–1.15)	0.98 (0.81–1.18)	1.11 (0.90–1.35)	1.15 (0.93–1.42)	0.004

*$p < 0.05$.

Model 1: unadjusted;

Model 2: adjusted for all covariates (number of covariates = 54) listed in Supplemental Table 2, except follow up duration, heart failure

medications during the admission, and heart failure medications within 3 months after discharge;
Model 3: adjusted for all the covariates listed in Supplemental Table 2 (number of covariates = 68), except follow up duration.
